# Intravoxel Incoherent Motion Model in Differentiating the Pathological Grades of Esophageal Carcinoma: Comparison of Mono-Exponential and Bi-Exponential Fit Model

**DOI:** 10.3389/fonc.2021.625891

**Published:** 2021-04-12

**Authors:** Nian Liu, Xiongxiong Yang, Lixing Lei, Ke Pan, Qianqian Liu, Xiaohua Huang

**Affiliations:** ^1^ Department of Radiology, Affiliated Hospital of North Sichuan Medical College, Nanchong, China; ^2^ Department of Radiology, Nanchong Hospital of Traditional Chinese Medicine, Nanchong, China

**Keywords:** esophageal squamous cell carcinoma, intravoxel incoherent motion, bi-exponential model, diffusion-weighted imaging, pathological grade, mono-exponential model

## Abstract

**Purpose:**

To compare the diagnostic efficiency of the mono-exponential model and bi-exponential model deriving from intravoxel incoherent motion diffusion-weighted imaging (IVIM-DWI) in differentiating the pathological grade of esophageal squamous cell carcinoma (ESCC).

**Methods:**

Fifty-four patients with ESCC were divided into three groups of poorly-differentiated (PD), moderately-differentiated (MD), and well-differentiated (WD), and underwent the IVIM-DWI scan. Mono-exponential (D_mono_, D*_mono_, and f_mono_) and bi-exponential fit parameters (D_bi_, D*_bi_, and f_bi_) were calculated using the IVIM data for the tumors. Mean parameter values of three groups were compared using a one-way ANOVA followed by *post hoc* tests. The receiver operating characteristic curve was drawn for differentiating pathological grade of ESCC. Correlations between pathological grades and IVIM parameters were analyzed.

**Results:**

There were significant differences in f_mono_ and f_bi_ among the PD, MD and WD ESCC groups (all p<0.05). The f_mono_ were 0.32 ± 0.07, 0.23 ± 0.08, and 0.16 ± 0.05, respectively, and the f_bi_ were 0.35 ± 0.08, 0.26 ± 0.10, and 0.18 ± 0.07, respectively. There was a significant difference in the D_mono_ between the WD and the PD group (1.48 ± 0.51* 10^-3^ mm^2^/s versus 1.05 ± 0.44*10^-3^ mm^2^/s, p<0.05), but there was no significant difference between the WD and MD groups, MD and PD groups (all p>0.05). The D*_mono_, D_bi_, and D*_bi_ showed no significant difference among the three groups (all p>0.05). The area under the curve (AUC) of D_mono_, f_mono_ and f_bi_ in differentiating WD from PD ESCC were 0.764, 0.961 and 0.932, and the sensitivity and specificity were 92.9% and 60%, 92.9% and 90%, 85.7% and 100%, respectively. The AUC of f_mono_ and f_bi_ in differentiating MD from PD ESCC were 0.839 and 0.757, and the sensitivity and specificity were 78.6% and 80%, 85.7% and 70%, respectively. The AUC of f_mono_ and f_bi_ in differentiating MD from WD ESCC were 0.746 and 0.740, and the sensitivity and specificity were 65% and 85%, 80% and 60%, respectively. The pathologically differentiated grade was correlated with all IVIM parameters (all p<0.05).

**Conclusions:**

The mono-exponential IVIM model is superior to the bi-exponential IVIM model in differentiating pathological grades of ESCC, which may be a promising imaging method to predict pathological grades of ESCC.

## Introduction

Diffusion-weighted imaging (DWI) is a quantitative technology for evaluating the water motion of tissues without injecting contrast agents (1). It is a mono-compartmental model of water diffusion, and signal attenuation is mono-exponential as a function of b value in traditional DWI (2, 3). Subsequently, le Bihan et al. (4) proposed a bi-exponential mathematical model that can surpass the traditional mono-compartmental model to quantify the effect of intravoxel incoherent motion (IVIM). IVIM-DWI has the advantage of separate evaluation of diffusion and perfusion changes in tissues (4). At present, the IVIM-DWI has been used to evaluate the pathological or blood perfusion status in the brain (5, 6) and abdominal organs (7–11). However, few studies (12–14) have been performed regarding the diagnosis and pathological grade of esophageal carcinoma with MRI, because conventional MRI is limited in its ability to resolve the early esophageal cancer. As the constant progress achieved in this field, IVIM-DWI demonstrated the potential value for the diagnosis and pathological grade of esophageal carcinoma (12, 14, 15).

Despite these promising studies, the accuracy and reliability of IVIM parameters are still challenged by a variety of fitting methods, such as the full fitting and segmented fitting method (16–19). The bi-exponential IVIM model adopts the full fitting method to reflect the organizational information of multi-component perfusion (3, 18). The mono-exponential IVIM model estimates IVIM parameters by the segmented fitting method, which was a classical IVIM model and different from the traditional DWI of mono-exponential diffusion model (3, 17, 18). The mono-exponential IVIM model is only suitable for the tissues with few perfusion components (3). Previous studies (17, 18) have suggested the accuracy and reliability of the mono-exponential IVIM model with segmented fitting are superior to the bi-exponential IVIM model with full fitting in the liver and the pancreas, but the full fitting method provided a better fit at very low and low b-values in the liver (18). Different fitting models have a different application for tissue components, especially for the prediction of different pathological differentiated tissues (20–22). To date, no studies have evaluated the different fitting models of IVIM-DWI for esophageal carcinoma. Therefore, it is still unclear whether the mono-exponential fit model or bi-exponential fit model in IVIM is suitable for esophageal carcinoma.

Therefore, the purpose of the present study is to determine whether the bi-exponential or mono-exponential fit model of IVIM can be used to distinguish the pathological grade of esophageal squamous cell carcinoma (ESCC), and which fitting model is more suitable for the pathological grade of ESCC.

## Materials and Methods

### Study Population

The study was approved by the ethics committee of the Affiliated Hospital of North Sichuan Medical College. Written informed consent was obtained from each participant. From January 2016 to February 2018, 68 consecutive patients with ESCC were enrolled in the present study according to the following inclusion criteria: patients with ESCC were confirmed by endoscopic pathology; and patients have not undergone any treatment for this disease before, such as radiotherapy, chemotherapy, and surgery; and MRI scan and IVIM-DWI were performed using the same magnetic resonance instrument. Fourteen patients were excluded, and the exclusion criteria were as follows: the patients had contraindications to MRI, or patients had a greater area of internal necrosis caused by lesions, or the images had severe motion artifacts. Finally, 54 cases of ESCC were included in this study.

The degree of pathological differentiation of the tumors was divided into poorly-differentiated (PD), moderately-differentiated (MD), and well-differentiated (WD) ESCC groups, and the T stage of tumors and tumor location (upper, middle, and lower esophagus) were determined according to the seventh edition guidelines of American Joint Committee on Cancer Stage (23).

### MR Imaging Techniques

MRI was performed using a 3.0T magnetic resonance instrument (Discovery MR 750, GE Medical Systems, Waukesha, WI, U.S.A.) with 32-channel phased-array body coil. Before the examination, patients were asked to fast for six hours and conduct shallow slow breath training, and to remove all metal objects. The patients were in a supine position and did not swallow during the examination. Patients were placed foot first, and supine with arms extended above their heads. The shimming was adopted to reduce gas interference before IVIM-DWI scanning. The respiratory-triggered technique and saturation suppression technology were used to avoid motion artifacts and guarantee the image quality of IVIM-DWI scans. The scanning parameters of IVIM-DWI are as follows: repetition time =6315.8 ms, echo time =58.2 ms, the thickness of layer 4 mm, interlay spacing 1.0 mm, the field of view =34 cm×34 cm, and matrix = 96×128. The ten b values were 0, 30, 50, 80, 150, 200, 400, 600, 800, 1000s/mm^2^, respectively. The corresponding number of excitations were 2, 2, 2, 2, 2, 2, 2, 4, 6, 8, respectively. The total scan time of IVIM-DWI was about 8 minutes.

Other sequences, such as the axial acquisition with volume acceleration-flexible T1-weighted imaging, respiratory-triggered axial propeller T2-weighted imaging (T2WI) with fat suppression, and axial single-shot fast spin-echo T2WI were also performed as routine work.

### Measurement and Calculation of Data

All the IVIM-DWI data were sent to GE Advantage Windows 4.5 Workstation for processing, using FuncTool software to obtain the ADC map in post-processing. IVIM-DWI and MADC maps were obtained after the images of adjacent fat, bone and gas were removed based on the definition of the diagnostic threshold. The region of interest (ROI) was drawn on the solid tumor components per MR image and three consecutive sections were selected by T2WI imaging for the measurement. Three ROIs were drawn on each section. Meanwhile, identification of a selection of the representative tumor tissue for ROI positioning was defined with the most cellularity part, and ROI was placed to cover as much of the solid part of the tumors as possible and to avoid cystic degeneration, necrosis, hemorrhage, and normal vessels to the greatest extent (12). The areas of ROI ranged from 53 mm^2^ to 55 mm^2^. Then, the software automatically generated the true diffusion coefficient (D) map, pseudo-diffusion coefficient (D*) map, and pseudo diffusion fraction (f) map and the corresponding parameters derived from the IVIM model. The deriving parameters of the mono-exponential model include D_mono_, D*_mono_, and f_mono_, and the deriving parameters of the bi-exponential model include D_bi_, D*_bi_, and f_bi_. It was measured by radiologists with 5 years of experience, and the average values of each parameter were taken.

IVIM parameters have a variety of calculation methods, including the Levenberg-Marquardt algorithm, segmentation constrained, bayesian probability, etc (16, 24). The Mono-exponential model used the segmentation constrained algorithm, and the bi-exponential model was fitted by nonlinear least-square based on the Levenberg-Marquardt algorithm (18). The mono-exponential model in IVIM adopted the method of asymptotic fitting, which can be obtained by using the fit equation [1] (18, 25):

[1] $$Sb/S0=f\;\times \exp^{\left(-bD*\right)}+\;\left(1-f\right)\times \exp^{\left(-bD\right)}$$

Sb is the signal intensity under a given b value, S0 is the signal intensity without diffusion weighting. The *D* represents the true diffusion coefficient, D* represents the pseudo-diffusion coefficient, and *f* represents the microvascular volume fraction.

The mono-exponential model IVIM-derived parameters used the segmentation constrained algorithm, which is divided into a high b-value part (usually b> 200 s/mm^2^) and a relatively low b-value part (usually b< 200 s/mm^2^) (18). When the b-value is large (>200 s/mm^–2^), since D* is significantly greater than D, the effects of D* on the signal attenuation can be ignored (4, 25, 26). So Eq. [1] can be simplified as follows:

[2] $$Sb/S0=\;\left(1-f\right)\;\times \exp^{\left(-bD\right)}$$

The curve of the high b-value data is considered as mono-exponential decay by equation [2], and the D value can be acquired with a simple linear fitting by equation [2]. The fitted curve was then extrapolated to get the intercept at b = 0. The f value was given through equation f= (S0-Sb)/S0 by the ratio of the intercept to the DWI data point at b = 0 (27). After substituting D and f value into the Equation [1] (26), D* values can be derived from the mono-exponential model with segmented fitting b-values.

The bi-exponential IVIM model is a full fitting of DWI signals to the bi-exponential function (18, 19, 28, 29). All b values are used to calculate IVIM parameters at the same time. First, high b values are used to calculate D values using Eq. [1]. Then, we fitted Sb for low b values using Eq. [1] after removing the effects of D value, and the f and D* simultaneously were obtained (28).

### Statistical Analysis

Statistical analyses were performed by SPSS22.0 software (Chicago, IL, USA) and MedCalc software Version 18.11 (MedCalcsoftware, Ostend, Belgium). Quantitative data were expressed in terms of mean ± standard deviation (¯x ± SD). The Shapiro-Wilks test was used to test the normality of distributions. If the data was a normal distribution, analysis of variance (ANOVA) was used to test the significant difference of IVIM parameters among three groups, and then *post hoc* with the least significant difference test was used. If the data was not a normal distribution, the Kruskal–Wallis test was used. Spearman’s rank correlation was used to analyze the correlation between IVIM parameters and pathological grade. Receiver Operating Curve (ROC) was drawn to evaluate the diagnostic efficiency of each parameter and to determine the diagnostic threshold value for the grading of ESCC. If the AUC is ranged from 0.5 to 0.7, then it is regarded as a low diagnostic value. If the AUC is ranged from 0.7 to 0.9, it is regarded as a moderate diagnostic value. If the AUC is greater than 0.9, it is regarded as a high diagnostic value. The two-tailed p-value less than 0.05 is considered statistically significant.

## Results

### General Characteristics of Patients

There were 54 patients with diagnosed ESCC (forty-three men and 11 women; mean age, 62.59 ± 6.65 years; age range, 49-76 years). There were 14 PD, 20 MD, and 20 WD ESCC by pathologically differentiated grade. There were 25 cases in stage T3, and 29 in stage T4. The tumors were located in the lower esophagus in 14 cases, the middle esophagus in 35, and the upper esophagus in 5.

### The Differences Between Mono-Exponential and Bi-Exponential Fitting Model Parameters Distinguishing the Pathological Grade of ESCC

The ANOVA tests showed that there were significant differences in D_mono_, f_mono,_ and f_bi_ among the WD group, MD group, and PD group (all p<0.05), whereas there was no difference in D*_mono_, D_bi_, and D*_bi_ (all p>0.05). Detailed results were shown in **Table 1**.

**Table 1:** The differences between the mono-exponential IVIM model and bi-exponential IVIM model distinguishing the pathological grade of ESCC ($\bar{x}$ ± SD).

Model parameters	WD (n=20)	MD (n=20)	PD (n=14)	Post-hoc by LSD			
	Mean ± SD	Mean ± SD	Mean ± SD	ANOVA	PD *vs* WD	PD *vs* MD	MD *vs* WD
				p value	p value	p value	p value
**Mono-exponential IVIM model**							
D_momo_ (10^-3^mm^2^/s)	1.48 ± 0.51	1.22 ± 0.39	1.05 ± 0.44	0.031^*^	0.010^*^	0.277	0.090
D*_mono_ (10^-2^mm^2^/s)	2.65 ± 1.96	1.88 ± 1.26	1.25 ± 0.56	0.147	NA	NA	NA
f_mono_	0.16 ± 0.05	0.23 ± 0.08	0.32 ± 0.07	<0.001^***^	<0.001^***^	<0.001^***^	<0.001^***^
**Bi-exponential IVIM model**							
D_bi_ (10^-3^mm^2^/s)	1.33 ± 0.54	1.17 ± 0.51	0.91 ± 0.45	0.065	NA	NA	NA
D*_bi_ (10^-2^mm^2^/s)	2.82 ± 1.63	2.21 ± 1.41	1.67 ± 0.78	0.059	NA	NA	NA
f_bi_	0.18 ± 0.07	0.26 ± 0.10	0.35 ± 0.08	<0.001^***^	<0.001^***^	0.005^**^	0.003**

^*^P < 0.05, ^**^P < 0.01, ^***^P < 0.001.

ESCC, esophageal squamous cell carcinoma; IVIM, intravoxel incoherent motion; D*, pseudo-diffusion coefficient; D, true diffusion coefficient; f, pseudo diffusion fraction; mono, mono-exponential fitting model; bi, bi-exponential fitting model; LSD, least significant difference; WD,well-differentiated; MD, moderately-differentiated; PD, poorly-differentiated.

**Figure 1:**
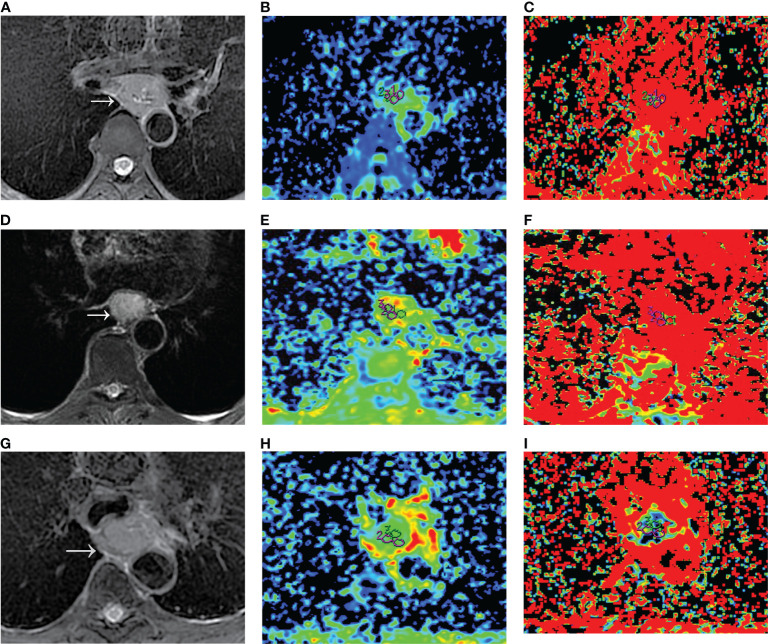
D_mono_ and f_mono_ values derived from mono-exponential IVIM-DWI of ESCC with different pathologically differentiated grades. **(A–C)** PD ESCC in a 48-year-old man. The regions of interest are selected by **(A)** T2-weighted image and drawn on **(B)** D_mono_ map (0.958×10^-3^ mm^2^/s) and **(C)** f_mono_ map (0.381×100%). **(D–F)** MD ESCC in a 64-year-old man. The regions of interest are selected by **(D)** T2-weighted image and drawn on **(E)** D_mono_ map (1.450×10^-3^ mm^2^/s) and **(F)** f_mono_ map (0.290×100%). **(G–I)** WD ESCC in a 61-year-old man. The regions of interest are selected by **(G)** T2-weighted image and drawn on **(H)** D_mono_ map (1.54×10^-3^ mm^2^/s) and **(I)** f_mono_ map (0.120×100%). IVIM, intravoxel incoherent motion; DWI, diffusion-weighted imaging; ESCC, esophageal squamous cell carcinoma; PD, poorly-differentiated; MD, moderately-differentiated; WD, well-differentiated; D, true diffusion coefficient; f, pseudo diffusion fraction.

For the mono-exponential fit parameters, there were significant differences in f_mono_ among the PD, MD, and WD groups (0.32 ± 0.07, 0.23 ± 0.08, and 0.16 ± 0.05, respectively; all p<0.05; **Figure 1**). A significant difference was found in the D_mono_ between the WD and the PD group (1.48 ± 0.51* 10^-3^ mm^2^/s versus 1.05 ± 0.44*10^-3^ mm^2^/s, p<0.05; **Figure 1**), but no significant difference was found between the WD and MD groups, MD and PD groups (1.48 ± 0.51* 10^-3^ mm^2^/s versus 1.22 ± 0.39*10^-3^ mm^2^/s, 1.22 ± 0.39*10^-3^ mm^2^/s versus 1.05 ± 0.44*10^-3^ mm^2^/s, respectively; all p>0.05).

For the bi-exponential fit parameters, there were significant differences in f_bi_ among the PD, MD, and WD ESCC groups (0.35 ± 0.08, 0.26 ± 0.10, and 0.18 ± 0.07, respectively; all p<0.05).

### The Diagnostic Efficacy Between the Two Models for the Pathological Grade of ESCC

**Figure 2:**
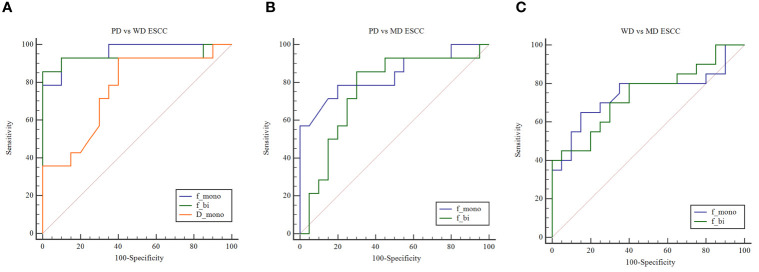
**(A)** ROC curves show the utility of D_mono_, f_mono,_ and f_bi_ to distinguish PD ESCC from WD ESCC, and **(B)** f_mono_ and f_bi_ to distinguish PD ESCC from MD ESCC, and **(C)** WD ESCC from MD ESCC. ROC, receiver operating characteristic; D, pure diffusion coefficient; f, perfusion fraction; ESCC, esophageal squamous cell carcinoma; PD, poorly-differentiated; MD, moderately-differentiated; WD, well-differentiated; mono, mono-exponential model; bi, bi-exponential model.

The area under curve (AUC) values of D_mono_, f_mono,_ and f_bi_ in differentiating WD from PD ESCC were 0.764, 0.961, and 0.932 (**Figure 2**). The sensitivity of D_mono_, f_mono,_ and f_bi_ was 92.9%, 92.9%, and 85.7%, and specificity was 60%, 90%, and 100%, respectively. The AUC value of f_mono_ and f_bi_ in differentiating MD from PD ESCC were 0.839 and 0.757 (**Figure 2**). The sensitivity of f_mono_ and f_bi_ was 78.6% and 85.7%, and specificity was 80% and 70%, respectively. The AUC of f_mono_ and f_bi_ in differentiating MD from WD ESCC were 0.746 and 0.740 (**Figure 2**). The sensitivity of f_mono_ and f_bi_ were 65% and 80%, and specificity was 85% and 60%, respectively. The AUC, sensitivity, specificity, and cut-off values of D_mono_, f_mono,_ and f_bi_ for differentiating pathological grade of ESCC were listed in **Table 2**.

**Table 2:** The diagnostic efficacy between the two models for the pathological grade of ESCC.

Differentiations	Variable	Cut-off value	AUC	Sensitivity	Specificity
PD *vs* WD					
	D_mono_	1.26*10^−3^ mm^2^/s	0.764	0.929	0.600
	f_mono_	0.214	0.961	0.929	0.900
	f_bi_	0.289	0.932	0.857	1.000
PD *vs* MD					
	f_mono_	0.301	0.839	0.786	0.800
	f_bi_	0.312	0.757	0.857	0.700
WD *vs* MD					
	f_mono_	0.193	0.746	0.650	0.850
	f_bi_	0.208	0.740	0.800	0.600

ESCC, esophageal squamous cell carcinoma; D, true diffusion coefficient; f, pseudo diffusion fraction; mono, mono-exponential fitting model; bi, bi-exponential fitting model; PD, poorly-differentiated; MD, moderately-differentiated; WD, well-differentiated.

### Correlations Between Mono-Exponential, Bi-Exponential Model Parameters, and Pathological Grade

For the mono-exponential model parameters, the pathologically differentiated grade correlated positively with the D_mono_ (r= 0.370, p=0.006) and D*_mono_ (r= 0.278, p= 0.042), and correlated negatively with f_mono_ values (r= -0.679, p< 0.001).

For the bi-exponential model parameters, the pathologically differentiated grade correlated positively with the D_bi_ (r= 0.489, p=0.001), D*_bi_ (r= 0.321, p= 0.018), and correlated negatively with f_bi_ values (r= -0.619, p< 0.001).

## Discussion

The present study evaluated the pathologically differentiated grade of ESCC by comparing the mono-exponential model and bi-exponential model in the post-processing of IVIM-DWI. The results demonstrated that the mono-exponential model parameters showed higher diagnostic value than the bi-exponential model parameters in differentiating pathologically grade of ESCC. Both the mono-exponential and bi-exponential fit parameter values correlated with pathologically differentiated grades. This finding provides a basis for the application of the IVIM-DWI model in the pathologically differentiated grade of ESCC. In our study, the mono-exponential model of IVIM was different from the traditional DWI model, and the traditional DWI with mono b value in these previous studies (4, 30–32) were used to detect the degree and direction of limitation in the *in vivo* water molecules movement. For reasons that capillary perfusion and the diffusion associated with microcirculation perfusion have a great effect on signal attenuation (4, 27), ADC of traditional mono-exponential diffusion model cannot reflect the diffusion of *in vivo* water molecules exactly and properly (33, 34). Previous studies (34–36) indicated that the IVIM-DWI model has higher accuracy than the traditional DWI with mono b value in the diagnosis of esophageal carcinoma. Therefore, our study compares the mono-exponential IVIM model and bi-exponential IVIM model parameters in differentiating the pathologically differentiated grade of ESCC rather than comparing the IVIM-DWI model and traditional mono-exponential DWI. These will help clinicians find a better fitting model for the pathologically differentiated grade of ESCC, and provide a reference for the selection of the optimal IVIM fitting model for tumor staging and evaluating prognosis.

In the present study, we found that the f_mono_ and D_mono_ value of mono-exponential IVIM model have higher diagnostic performance for differentiation of ESCC, whereas the bi-exponential IVIM model only had f_bi_ values. One of the possible reasons is that the mono-exponential model used the segmentation constrained algorithm, and the bi-exponential model used a full fitting based on the Levenberg-Marquardt algorithm. Though both the two fitting methods have similar repeatability (17). Previous studies (18, 19) have proved that the mono-exponential fitting model demonstrated a more accurate estimation of D in signal prediction for high b-values in the abdomen relative to the bi-exponential fitting model, but tending to underestimate D* (18). The bi-exponential fitting model should allow more flexibility and provided a better fit and a more accurate estimation of D* at low b-values (18), so it is possible to produce results closer to the true physiological value of IVIM parameters. Therefore, the different fitting methods of the two models in our study may explain the difference. The second possibility is that the mono-exponential IVIM model may detect one or a few perfusion components, while the bi-exponential IVIM model reflects the organizational information of multi-component perfusion (3). A collection of vessels with similar physiological properties can be usually regarded as one perfusion component, such as vascular size, blood flow velocity, and vascular spatial configuration (3, 37). Therefore, it can be inferred that the mono-exponential IVIM model could more truly reflect the perfusion component and diffusion information of esophageal carcinoma, and the mono-exponential IVIM model may be more suitable for predicting the pathological grade of ESCC.

It is noteworthy that both the f_mono_ and f_bi_ can help differentiate PD, MD, and WD ESCC. The mean f_mono_ and f_bi_ values decreased gradually from the PD group to the WD group. The f_mono_ and f_bi_ were correlated negatively with the pathologically differentiated grade of ESCC. The previous study (12) supported our results, and they also reported that the f value was a gradually decreasing trend from the PD group to the WD group. Still, f values showed no statistical difference among the three groups. The f value represents microcapillary perfusion and reflects the vascularity in tissue, which is helpful to assess the pathological differentiation grade of the tumor (28, 38). The f value was positively correlated with microvessel density (39). Previous studies (40, 41) have reported that the microvessel density of moderately- or well-differentiated esophageal carcinoma was lower than that of poorly-differentiated esophageal carcinoma. These findings further support our study. Therefore, both the f_mono_ and f_bi_ can be used in differentiating well-differentiated, moderately-differentiated, and poorly-differentiated ESCC.

Our study also demonstrated that both the D*_mono_ and D*_bi_ were not statistically significant in differentiating the PD, MD, and WD ESCC, which was similar to previous studies (12, 42). Previous studies (3, 16, 27, 43) have shown that the D* often suffers from high variance and standard deviation, which can obscure or misinterpret pathologies in clinical applications. The parameter variance of the IVIM model is easy to be disturbed by many factors (16, 44). For example, they are susceptible to four major factors (3, 17, 45, 46), such as the noise in DWI, the different b values, the fitting techniques or model, and the artifact of cardiac and breathing motions. However, there is no uniform standard for the selection of b values in clinical practice so far. So long as the distribution of b values is reasonable, the parameters of the IVIM model may be no longer affected by b values (7, 45). At the same time, we avoided and excluded the artifacts of cardiac and breathing motions by respiratory gating and shimming. Also, the D* value is associated with blood flow velocity and vascular length (3). The blood supply of the esophageal tumors varies greatly, which is mainly related to the anatomical location of the esophagus (12) and may influence the perfusion of the tumor. Therefore, D* was not useful for the evaluation of the pathological differentiation grade of ESCC.

In our study, the pathologically differentiated grade of ESCC was correlated negatively with the f_mono_ and f_bi_ and correlated positively with the D_mono_, D*_mono_, D_bi_, and D*_bi_, which indicated that the parameters derived from IVIM-DWI could well reflect the differentiation grade of esophageal carcinoma. The lower the degree of tumor differentiation pathologically, the larger the cell atypia and the tumor cell density (28). With the lower the degree of differentiation of esophageal carcinoma, the increase of tumor cell density will lead to a more limited spread and finally lead to the decrease of D value (28). The differentiation grade of ESCC is an important prognostic indicator (47, 48), and it is also one of the indicators to choose the best therapeutic alternative (49, 50). Therefore, IVIM-DWI could be a promising and non-invasive imaging method in predicting the pathological grade of ESCC.

There are several limitations to this study. First, the sample size was relatively small, especially in the poorly differentiated ESCC. And the sample lack T1 and T2 stage ESCC, because most patients don’t have any symptoms until the T3 or T4 stage. We will enlarge the sample size to analyze the correlation between the two IVIM models and the stage in the further. Second, we did not compare mono-exponential IVIM and bi-exponential IVIM models between different pathological types of esophageal cancers, but samples containing only ESCC eliminated other confounding factors for the findings. Therefore, we need larger different pathological types of sample size in further studies. Third, the correlation between the tumor’s location and two IVIM models were not investigated. Fourth, the accuracy of the parameters may differ according to the different b-value ranges for target lesions. But the analysis of signal intensities averages over an ROI approach and segmented fitting method combined with the reasonable b value distributions could lead to considerable improvement in accuracy (17, 45). Finally, esophageal peristalsis or glandular secretion can cause signal decay, which may be difficult to differentiate from perfusion effects (15). Though we ask the patient not to swallow during the examination and use the respiratory-triggered and saturation suppression technique, these issues need to be considered when interpreting our findings. Despite these limitations, the present study depicted the difference between mono-exponential IVIM and bi-exponential IVIM fitting models in the evaluation of pathological differentiation grade of ESCC.

## Conclusion

In conclusion, the f_mono_ derived from mono-exponential IVIM-DWI shows higher diagnostic performance than f_bi_ derived from bi-exponential IVIM-DWI in differentiating WD, MD, and PD ESCC, and D_mono_ derived from mono-exponential IVIM-DWI can distinguish PD from WD ESCC. The mono-exponential fit parameters derived from IVIM are superior to the bi-exponential fit parameters in differentiating pathologically differentiated grades of ESCC, which may be a promising non-invasive imaging method to predict the pathological grade of ESCC. The findings may help to select an appropriate fitting model for the application of IVIM in ESCC and improve the diagnostic accuracy.

## Data Availability Statement

The raw data supporting the conclusions of this article will be made available by the authors, without undue reservation.

## Ethics Statement

The studies involving human participants were reviewed and approved by Affiliated Hospital of North Sichuan Medical College. The patients/participants provided their written informed consent to participate in this study.

## Author Contributions

NL, XY and LL collected the cases and analyzed the data. KP and QL performed the MRI scan. NL and XY wrote the manuscript, and XH made substantial contributions to the conception and revised the manuscript. All authors contributed to the article and approved the submitted version.

## Funding

This study was supported by the Bureau of Science & Technology and Intellectual Property Nanchong City (NO. 19SXHZ0429, XH), and the Science and Technology Development Plan of North Sichuan Medical College (No. CBY14-A-ZD06, NL).

## Conflict of Interest

The authors declare that the research was conducted in the absence of any commercial or financial relationships that could be construed as a potential conflict of interest.

## Abbreviations

IVIM, intravoxel incoherent motion; DWI, diffusion-weighted imaging; ESCC, esophageal squamous cell carcinoma; PD, poorly-differentiated; MD, moderately-differentiated; WD, well-differentiated; D, true diffusion coefficient; D*, pseudo-diffusion coefficient; f, pseudo diffusion fraction; mono, mono-exponential fitting; bi, bi-exponential fitting; T2WI, T2-weighted imaging; ROC, Receiver Operating Curve; AUC, area under curve.
